# Toll-Like Receptor 3 and Suppressor of Cytokine Signaling Proteins Regulate CXCR4 and CXCR7 Expression in Bone Marrow-Derived Human Multipotent Stromal Cells

**DOI:** 10.1371/journal.pone.0039592

**Published:** 2012-06-22

**Authors:** Suzanne L. Tomchuck, Sarah L. Henkle, Seth B. Coffelt, Aline M. Betancourt

**Affiliations:** 1 Department of Microbiology & Immunology, Tulane University Health Sciences Center, New Orleans, Louisiana, United States of America; 2 Tulane Center for Stem Cell Research and Regenerative Medicine, Tulane University Health Sciences Center, New Orleans, Louisiana, United States of America; 3 Division of Molecular Biology, The Netherlands Cancer Institute, Amsterdam, The Netherlands; Friedrich-Alexander University Erlangen, Germany

## Abstract

**Background:**

The use of bone marrow-derived human multipotent stromal cells (hMSC) in cell-based therapies has dramatically increased in recent years, as researchers have exploited the ability of these cells to migrate to sites of tissue injury, inflammation, and tumors. Our group established that hMSC respond to “danger” signals – by-products of damaged, infected or inflamed tissues – via activation of Toll-like receptors (TLRs). However, little is known regarding downstream signaling mediated by TLRs in hMSC.

**Methodology/Principal Findings:**

We demonstrate that TLR3 stimulation activates a Janus kinase (JAK) 2/signal transducer and activator of transcription (STAT) 1 pathway, and increases expression of suppressor of cytokine signaling (SOCS) 1 and SOCS3 in hMSC. Our studies suggest that each of these SOCS plays a distinct role in negatively regulating TLR3 and JAK/STAT signaling. TLR3-mediated interferon regulatory factor 1 (IRF1) expression was inhibited by SOCS3 overexpression in hMSC while SOCS1 overexpression reduced STAT1 activation. Furthermore, our study is the first to demonstrate that when TLR3 is activated in hMSC, expression of CXCR4 and CXCR7 is downregulated. SOCS3 overexpression inhibited internalization of both CXCR4 and CXCR7 following TLR3 stimulation. In contrast, SOCS1 overexpression only inhibited CXCR7 internalization.

**Conclusion/Significance:**

These results demonstrate that SOCS1 and SOCS3 each play a functionally distinct role in modulating TLR3, JAK/STAT, and CXCR4/CXCR7 signaling in hMSC and shed further light on the way hMSC respond to danger signals.

## Introduction

Human MSC are excellent vehicles for cell-based therapeutics because they are easily isolated and can be exponentially expanded *ex vivo*. The use of hMSC in cell-based therapies has dramatically increased in recent years, as researchers have also exploited the ability of these cells to migrate to sites of tissue injury, inflammation, and tumors. Numerous studies using hMSC transplants have shown that these cells improve the outcome of disease, especially autoimmune disorders [1], [2], [3], [4], [5], [6], [7], due to their immunomodulatory properties [8], [9], [10]. In order to enhance hMSC-based therapeutics, it is imperative to understand the mechanisms by which hMSC are recruited to the target site, since less than 0.1% of these cells engraft into the tissue [11], [12], [13], [14].

Our group and others have demonstrated that MSC respond to “danger” signals – by-products of damaged, infected or inflamed tissues – via activation of TLR signaling [15], [16], [17]. TLRs have been shown to modulate proliferation, differentiation and migration of MSC, but these functional responses largely depend on both the tissue and species from which the MSC were derived [15], [16], [17], [18], [19]. Our group previously reported that activation of TLR signaling produces a unique gene expression profile in hMSC depending on the agonist with which they are treated. In addition, stimulation of TLR3 led to unique responses from hMSC including enhanced migratory ability. Exposure of hMSC to TLR3 ligands may represent a distinct mechanism through which these cells sense danger signals and preferentially migrate to the target tissue in order to perform their reparative function. Furthermore, studies from our group and others have also linked TLR activation to the regulation of immunomodulatory factors in hMSC, including indoleamine 2,3-dioxygenase and prostaglandin E_2_ [18], [19]. Taken together, emerging evidence indicates that TLRs affect important biological functions of hMSC, including migration, differentiation, and immunomodulation.

Although mounting evidence shows that TLRs contribute to MSC function, little is known of what happens downstream of the TLR signaling pathway, especially what factors may negatively regulate TLR signaling and thus may also modulate MSC function. Suppressor of cytokine signaling (SOCS) proteins are a well established family of E3 ubiquitin ligases which can be induced directly through TLR-mediated gene expression, or indirectly by the expression of TLR-induced chemokines and cytokines that activate the JAK/STAT pathway [20]. Several studies have identified SOCS1 as a negative regulator of TLR signaling by targeting TLR adaptors [21], intermediate signaling components [22] and TLR-activated transcription factors [23] for degradation or by inhibiting phosphorylation. The vast majority of TLR-SOCS studies have centered on lipopolysaccharide-induced TLR4 signaling and the role of SOCS1 as a negative regulator. However, Hashimoto and colleagues found that polyinosinic:polycytidylic acid (poly(I:C))-induced TLR3 signaling indirectly induced SOCS1 expression in human keratinocytes, which inhibited both TLR3-mediated STAT1 activation and chemokine expression [24]. Conversely, Yang and colleagues found that poly(I:C)-induced TLR3 activation resulted in the upregulation of SOCS3 in murine dendritic cells, which inhibited TLR3 indirectly by promoting degradation of tyrosine kinase 2 [25].

Furthermore, SOCS proteins have also been identified as negative regulators of chemokine signaling by associating with the receptor and inhibiting activation of signaling and function, such as migration [26], [27]. The Mellado group determined that SOCS3 upregulation in hematopoietic stem cells inhibited their retention in the bone marrow, a function primarily regulated by CXCL12 expression [28], by inhibiting its receptor, CXCR4 [29]. Additionally, the Lider group examined how TLR activation negatively regulates CXCR4 signaling through activation of SOCS expression; TLR2 signaling inhibited CXCR4-mediated T cell homing in a SOCS3-dependent mechanism [30].

Since the SOCS family is known to regulate various aspects of immune cell function, including differentiation, proliferation and migration [31], we hypothesized that SOCS may also play a role in regulating TLR- and CXCR-mediated signaling within hMSC [32], [33], [34], [35], [36], [37].

In this study we determined that SOCS1 and SOCS3 each play a unique role in negatively regulating TLR3-mediated signaling in hMSC. The expression of SOCS1 inhibited JAK2/STAT1 signaling, while SOCS3 inhibited IRF1 signaling. We also demonstrate for the first time that TLR3 signaling reduced cell surface expression of both CXCR4 and CXCR7 by receptor internalization and degradation in hMSC, which was disrupted when SOCS were overexpressed. Collectively, these data suggest that as negative regulators, SOCS proteins affect the way hMSC function and thus may also play a pivotal role in hMSC immune regulation with critical implications for hMSC-based therapies.

## Materials and Methods

### Human Multipotent Stromal Cells

Bone marrow-derived hMSC were used for all studies (Tulane Center for Stem Cell Research and Regenerative Medicine, New Orleans, LA; Lonza, Walkersville, MD). Human MSC are pre-tested for their homogeneity and differential potential to chondro-, osteo-, and adipogenic lineages by the suppliers. Our lab also verified all hMSC were positive for CD90, CD105, CD106, CD164, CD56, CD166, CD29, and CD44, and negative for CD45, CD14, CD31, CD34, HLA DR and CD117 by flow cytometry. Human MSC were cultured as previously described [17]. All experiments were conducted on hMSC at a passage ≤5 with at least three different donors from the two differences sources stated above.

### Transfection and Vectors

Human MSC were transfected by electroporation using the Invitrogen Neon system (Carlsbad, CA) according to the manufacturer’s instructions using 2.5 µg vector per 1×10^6^ cells. Human MSC were transfected with the SOCS overexpression vectors, pORF5-hSOCS1 or pORF5-hSOCS3, or mock vector, pORF5-MCS as indicated (Invivogen, San Diego, CA). Overexpression was confirmed 24 hours post transfection by Western blot analysis. All transfected cells were allowed to recover overnight before experimental treatments. Cell viability measured for the hMSC following transfection was typically 90%. Transfection efficiency with the optimized MSC protocol for Neon System was typically 80% (Invitrogen).

### TLR3 Ligand

To induce TLR3 activation, hMSC were treated with 10 µg/ml of poly(I:C), a dsRNA analog (InvivoGen).

### Human JAK/STAT Signaling Pathway RT^2^ Profiler™ PCR Array

Human MSC were treated for 6 hours with poly(I:C), washed twice, RNA was extracted using the RNeasy Mini Kit (Qiagen, Valencia, CA), and then treated using the TURBO DNA-free kit (Ambion, Austin, TX). RNA was reverse transcribed and the resulting cDNA was used in the JAK/STAT Signaling Pathway RT^2^ Profiler™ PCR Array (SuperArray Bioscience, Frederick, MD) according to the manufacturer’s instructions on an iCycler iQ5 Real-Time PCR Detection System (Bio-Rad, Hercules, CA). Raw data from both the untreated and treated groups were analyzed using the GEarray Analyzer software (SuperArray Inc., Bethesda, MD).

### Quantitative Real-Time PCR (qPCR)

Human MSC were treated for 6 hours with poly(I:C), RNA was extracted using the RNeasy Mini Kit (Qiagen,), and then treated using the TURBO DNA-free kit (Ambion). RNA was subsequently reverse transcribed using the iScript™ cDNA Synthesis kit according to the manufacturer’s instructions (Bio-Rad). cDNA was amplified in iQ SYBR Green Supermix (Bio-Rad) with the following primers pairs: *IRF1* - Forward 5′-CTT CCA CCT CTC ACC AAG AAC-3′, Reverse 5′-CCA TCA GAG AAG GTA TCA GGG C-3′; *JAK2* - Forward 5′-AAG AAA ACG ATC AAA CCC CAC T-3′ Reverse 5′-TGC ATT GGC TGA ATT GCT GAA-3'; *SOCS1* - Forward 5′-GCC TGC GGA TTC TAC TGG G-3', Reverse 5′-TAA GGG CGA AAA AGC AGT TCC-3'; *18S rRNA -* Forward 5′-GAG GGA GCC TGA GAA ACG G-3′, Reverse 5′-GTC GGG AGT GGG TAA TTT GC-3′ (IDT, Coralville, IA), using the iCycler iQ5 Real-Time PCR Detection System (Bio-Rad). Optimal primer efficiencies and cDNA concentrations were determined before conducting real-time PCR. As a reference gene, *18S rRNA* specific primers were used. The experimental samples and internal controls were run in triplicate on the same plate. The qPCR reaction was carried out as previously described [38]. Differences in gene expression were determined by the Quantitative Comparative C_T_ (threshold value) method. qPCR was conducted using 5 separate RNA isolations from 5 different hMSC donors.

### Western Blot Analysis

Human MSC were treated with poly(I:C) to induce TLR3 activation as indicated. Protein was isolated and concentration measured as previously described [17]. Treatment was carried out in triplicate per donor and then pooled together during protein isolation. Approximately 20 µg of protein was then separated by electrophoresis on a NuPage Novex 4–12% BIS-TRIS polyacrylamide gel (Invitrogen). Proteins were then transferred onto a nitrocellulose membrane using the Invitrogen iBlot system. Membranes were blocked for 1 hour with 5% non-fat dry milk in Dulbecco’s phosphate buffered saline containing 0.2% Tween-20 (DPBST). pJAK2^Y1007/1008^, JAK2 (rabbit monoclonal antibodies; Cell Signaling Technology, Beverly, MA), pSTAT1^Y701^, STAT1, pSTAT3^Y705^, STAT3, pSTAT5^Y694^, STAT5 (mouse monoclonal antibodies; BD Biosciences, San Jose, CA), IRF-1, SOCS1, SOCS3, CXCR4, or CXCR7 (rabbit polyclonal antibodies; Abcam, Cambridge, MA) primary antibodies were diluted in DPBST and incubated overnight at 4°C. After washing 3 times in DPBST, membranes were incubated for 1 hour in an HRP-conjugated secondary antibody diluted (Amersham, Piscataway, NJ) in DPBST. Membranes were washed again, and immunodetection of the protein was carried out using ECL (Invitrogen) and imaged by the FUJI LAS-4000 Imager (Tokyo, Japan). Restore Plus Stripping Buffer (Thermo Scientific, Waltham, MA) was used to strip membrane, which were re-blocked and incubated with a β-actin primary antibody as a normalizing control (Sigma-Aldrich, St. Louis, MO). Each Western blot was repeated a minimum of 3 times, each time with a different donor. Band densitometry was determined using ImageJ software. After subtracting overall background, experimental bands were first normalized to the actin loading control band within each lane, and then expressed as fold change from the untreated control band. Data displayed are representative of the results obtained from 3 separate donors.

### Flow Cytometry

Human MSC were treated with poly(I:C) for 0, 2, 4, or 6 hours, harvested and analyzed by flow cytometry as previously described using a CXCR4 or CXCR7 primary antibody and an Alexa-488-conjugated secondary antibody (Invitrogen) [17]. Samples were run on a BD FACSCalibur and analyzed using CellQuest Pro software (BD Biosciences). Flow cytometry analysis was performed using a minimum of 3 different hMSC donors and unstained cells and isotype antibody controls served as negative controls. Statistical significance was determined as follows: after subtracting the mean fluorescence intensity (MFI) of the isotype control, the MFI of each experimental histogram was expressed as fold change from the untreated control.

### Immunofluorescence Assay

Human MSC were plated onto tissue culture treated slides, 1.5×10^4^ cells/chamber and treated with poly(I:C) for 0, 2, 4 or 6 hours. Fluorescence immunocytochemistry was then performed as previously described using a CXCR4 (Abcam, Invitrogen) and/or CXCR7 (Abcam) primary antibody with an Alexa-488 conjugated secondary antibody, and the nucleus was stained with 15 nM 4',6-diamidino-2-phenylindole (DAPI; Sigma) [17]. A minimum of 5 different human MSC donors from two sources was assayed in duplicate. Unstained cells and cells stained with an Alexa-488 conjugated secondary antibody alone served as negative controls. Slides were analyzed using a Zeiss Axio Plan II microscope and SlideBook Version 5 Software. All photomicrographs were taken with a 40X objective. Colocalization was determined by both the Pearson’s and Manders’ coefficient using the ImageJ software plugin, JACoP v2.0 [39].

### Statistical Analysis

Data is presented as the mean ± the standard error of the mean (SEM). Data were analyzed by comparing the treated to untreated groups by a two-tailed, unpaired t-test (GraphPad Prism Version 4). P-values below 0.05 were considered statistically significant.

## Results

### TLR3 Stimulation Activates a JAK2/STAT1 Pathway in hMSC

#### TLR3 stimulation alters the expression of JAK/STAT-related genes

In order to determine what components of the JAK/STAT pathway may be affected by TLR3 stimulation, we treated hMSC with the TLR3 agonist, poly(I:C), and performed a human JAK/STAT Signaling Pathway RT^2^ Profiler™ PCR Array. Among the 84 different JAK/STAT-related genes, we found that *JAK2* was elevated 9.85-fold, the transcription factor *IRF1* was upregulated 16-fold, and *SOCS1* was upregulated 21.11-fold; several other genes were also induced or inhibited at least 2-fold following TLR3 stimulation (**Table 1**).

**Table 1:** TLR3 stimulation alters the expression of JAK/STAT-related genes.

Gene	Fold Change	Gene	Fold Change
Alpha-2-macroglobulin	2.30	V-myc myelocytomatosis viral oncogene homolog	−2.30
Coagulation factor II (thrombin) receptor	−3.25	Nuclear factor of kappa 1 (p105)	2.83
Colony stimulating factor 2 receptor, beta	−2.30	Nitric oxide synthase 2A (inducible, hepatocytes)	8.00
C-X-C chemokine ligand 9	3821.70	2',5'-oligoadenylate synthetase 1	111.43
Epidermal growth factor receptor	2.14	Platelet-derived growth factor receptor, alpha polypeptide	−2.30
Erythropoietin receptor	−2.46	STAT2	−3.25
Fas (Tumor necrosis factor receptor superfamily, member 6)	2.64	SH2B adaptor protein 1	−2.46
Fc fragment of immunoglobulin G, high affinity Ia,receptor (CD64)	2.83	Signal transducing adaptor molecule 1	−2.30
Guanylate binding protein 1, interferon-inducible, 67kDa	36.76	SOCS1	21.11
IRF1	16.00	STAT2	3.48
Interferon-stimulated transcription factor (ISG) 3, gamma	2.14	STAT4	2.64
Interleukin 2 receptor, gamma	6.50	STAT5A	4.59
ISG15 ubiquitin-like modifier	51.98	Upstream transcription factor 1	2.83
JAK2	9.85	YY1 transcription factor	2.64
Matrix metallopeptidase 3	13.00		

RNA was isolated from hMSC treated for 6 hours with poly(I:C) and analyzed for 84 genes relating to JAK/STAT signaling using an RT^2^ PCR array. Only genes with a greater than 2-fold increase or decrease in expression are displayed.

**Figure 1:**
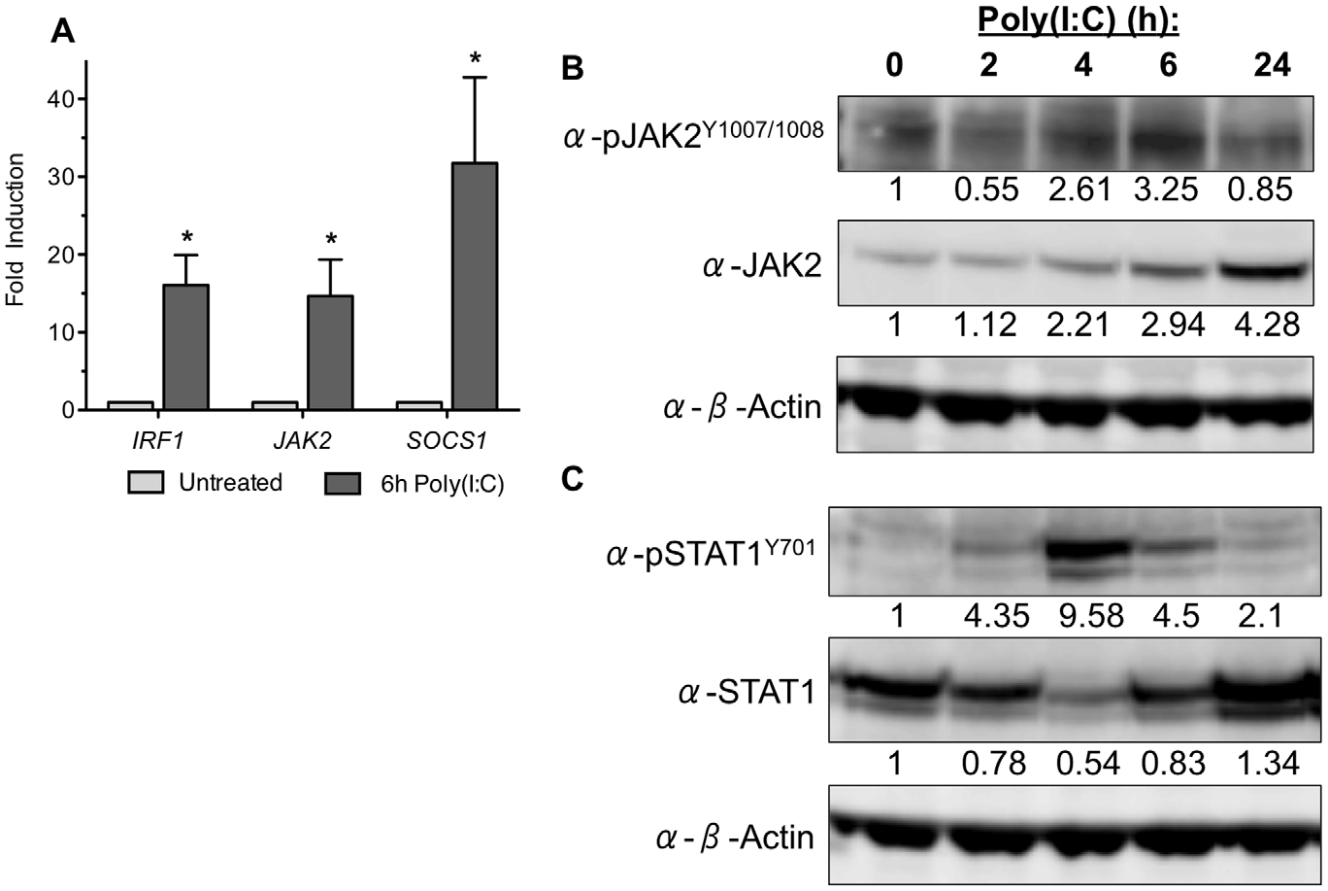
TLR3 stimulation activates a JAK2/STAT1 pathway in hMSC. (A) Human MSC gene induction, following 6 hours of poly(I:C) treatment, was analyzed by qPCR using 5 separate donors. Data are presented as mean ± SEM. Significance was determined by comparing the poly(I:C) treated samples to the untreated control. *p<0.05. (B-C) JAK2/STAT1 phosphorylation was analyzed by Western blot following TLR3 stimulation for 0–6 hours. Of note, the monoclonal STAT1 antibody preferentially binds total STAT1, which is why total STAT1 expression appears inversely proportional to phosphorylated STAT1. Densitometry was determined by subtracting overall background, then each experimental band was normalized to the actin loading control band within its lane, and fold change was calculated based upon the untreated control band. Density values below each band are representative of results from 3 separate donors.

In order to verify these results, qPCR was performed on a select number of genes that showed at least a two-fold increase or decrease in mRNA expression after poly(I:C) treatment. TLR3 activation significantly induced *IRF1* (16.08±3.86), *JAK2* (14.68±4.67), and *SOCS1* (31.74±11.05) (**Figure 1A**). These results follow the same trend as the JAK/STAT PCR array, suggesting that TLR3 activation results in downstream signaling of the JAK/STAT pathway.

#### JAK2 is upregulated and activated upon TLR3 stimulation

Given the induction of *JAK2* mRNA following TLR3 stimulation we next examined the effect of poly(I:C) on JAK2 enzyme activation and expression. JAK2 phosphorylation was evident 4 to 6 hours after poly(I:C) treatment of hMSC. In addition, total expression levels of JAK2 steadily increased beginning at the same time point (**Figure 1B**), further supporting our qPCR results. Interestingly, TLR3 activation and subsequent downstream signaling pathway activation was specific for JAK2, since JAK1 and JAK3 were not phosphorylated following poly(I:C) treatment (data not shown).

#### STAT1 is activated after 4 hours of TLR3 stimulation

After establishing that JAK2 is activated by TLR3 stimulation we wanted to investigate which STATs were subsequently activated to carry out the signaling pathway. STAT1 phosphorylation in hMSC treated with poly(I:C) showed a narrow window of activation, between 2 and 6 hours of treatment. After 4 hours of stimulation, STAT1 activation peaked (**Figure 1C**). While total STAT1 appears to have an inversely proportional expression pattern as that of phosphorylated STAT1, this is only an artifact due to the nature of the mouse monoclonal antibody used, which preferentially binds to unphosphorylated STAT. By contrast, Western blot analyses of phosphorylated STAT3 and STAT5, using mouse monoclonal antibodies, showed that TLR3 stimulation was unable to activate either STAT; neither STAT3 nor STAT5 showed any changes in phosphorylation following treatment (**Figure S1**). Collectively, these experiments suggest that TLR3-mediated downstream signaling in hMSC is carried out by JAK2 and STAT1.

### SOCS Overexpression Disrupts STAT1 Activation

#### SOCS1 expression is altered following TLR3 activation

**Figure 2:**
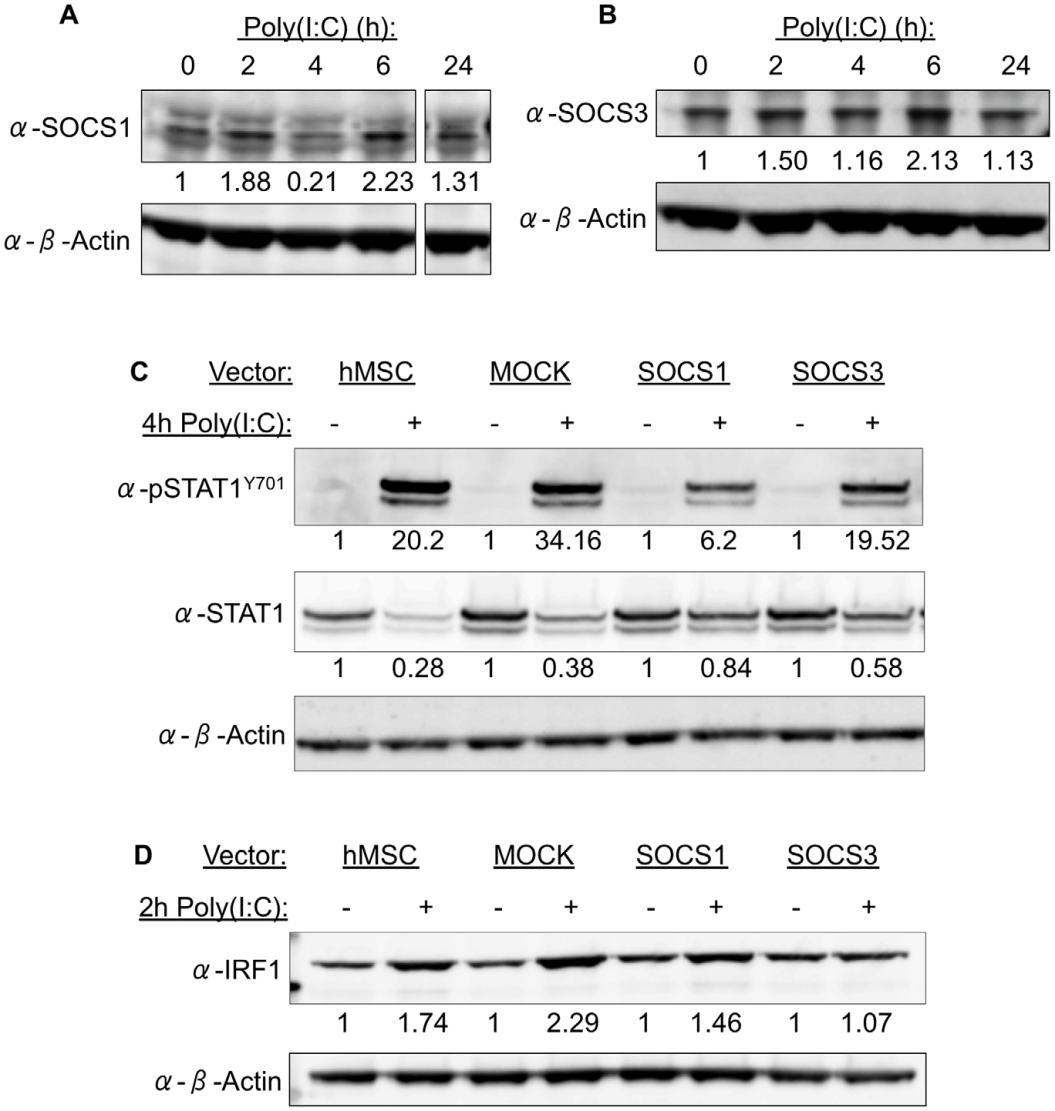
SOCS overexpression disrupts STAT1 activation. (A-B) SOCS1 and SOCS3 expression following TLR3 activation in hMSC by Western blot analysis. (C) STAT1 activation in untransfected, mock-transfected, SOCS1- or SOCS3-overexpressing hMSC after 4 hours of poly(I:C) treatment. (D) IRF1 expression in untransfected, mock-transfected, SOCS1- or SOCS3-overexpressing hMSC after 2 hours of poly(I:C) treatment. Density values below each band are representative of results from 3 separate donors.

SOCS proteins are negative regulators of both JAK/STAT and TLR signaling [20], [22]. After establishing that TLR3-mediated signaling resulted in an upregulation of *SOCS1* mRNA (**Figure 1A**), we then examined how TLR3 activation affected SOCS1 protein expression. TLR3 stimulation did alter SOCS1 expression, with an induction at 2 and 6 hours of treatment, and reduced expression at 4 hours (**Figure 2A**). These Western blot data suggest that during TLR3 activation, SOCS1 tightly regulates STAT1 phosphorylation and only allows for its activation for a short period of time. SOCS1 expression was inversely proportional to STAT1 phosphorylation, where reduced SOCS1 expression at 4 hours of treatment allowed for peak STAT1 phosphorylation. When SOCS1 expression then increased at 6 hours of treatment, STAT1 activation was then diminished.

#### SOCS3 expression is also affected following TLR3 activation

Although our JAK/STAT Signaling Pathway RT^2^ Profiler™ PCR Array did not show any significant changes in *SOCS3* mRNA expression (data not shown), previous studies have demonstrated that like SOCS1, SOCS3 also plays a role in modulating both JAK/STAT and TLR signaling [20], [22], [40]. We therefore decided to explore SOCS3 protein expression in hMSC after TLR3 activation. At 6 hours of treatment, SOCS3 expression, like SOCS1, was also upregulated (**Figure 2B**).

#### SOCS1 overexpression inhibits STAT1 activation

We then tested if either SOCS1 or SOCS3 was able to disrupt TLR3 signaling. As dsRNA is an agonist of the TLR3 pathway, we were unable to perform knockdown studies using shRNA or siRNA to inhibit SOCS1 or SOCS3, which would activate rather than inhibit TLR signaling [17], [19], [41], [42]. Given that confounding potential, we chose to overexpress these SOCS and studied their effects on the TLR3 pathway in this manner. hMSC were transfected with either a SOCS1 or SOCS3 overexpression vector, allowed to recover overnight, and then TLR3 stimulated. We found that the untransfected, mock-transfected, and SOCS3-overexpressing hMSC all showed robust STAT1 phosphorylation after 4 hours of poly(I:C) treatment (**Figure 2**C). The SOCS1-overexpressing hMSC, however, had diminished levels of STAT1 phosphorylation, demonstrating that only the overexpression of SOCS1 was able to inhibit STAT1 activation. These results support our previous Western blots of untransfected hMSC, where the upregulation of SOCS1 at 6 hours of treatment correlated to a decrease in STAT1 phosphorylation, thereby impeding the signaling pathway.

#### SOCS3 overexpression inhibits IRF1 upregulation

Furthermore, when examining IRF1 expression after TLR3 activation, we found that IRF1 was upregulated after 2 hours of treatment in untransfected, mock-transfected, and SOCS1-overexpressing cells (**Figure 2D**). In contrast, SOCS3-overexpressing hMSC showed no IRF1 upregulation after TLR3 activation.

Our results showed that SOCS1 reduced STAT1 activation while SOCS3 inhibited IRF1 upregulation, which indicate that SOCS1 and SOCS3 each play a distinct role in regulating TLR3 signaling in hMSC.

### TLR3-mediated Internalization and Degradation of CXCR4 and CXCR7

#### TLR3 activation leads to attenuated cell surface expression of CXCR4

**Figure 3:**
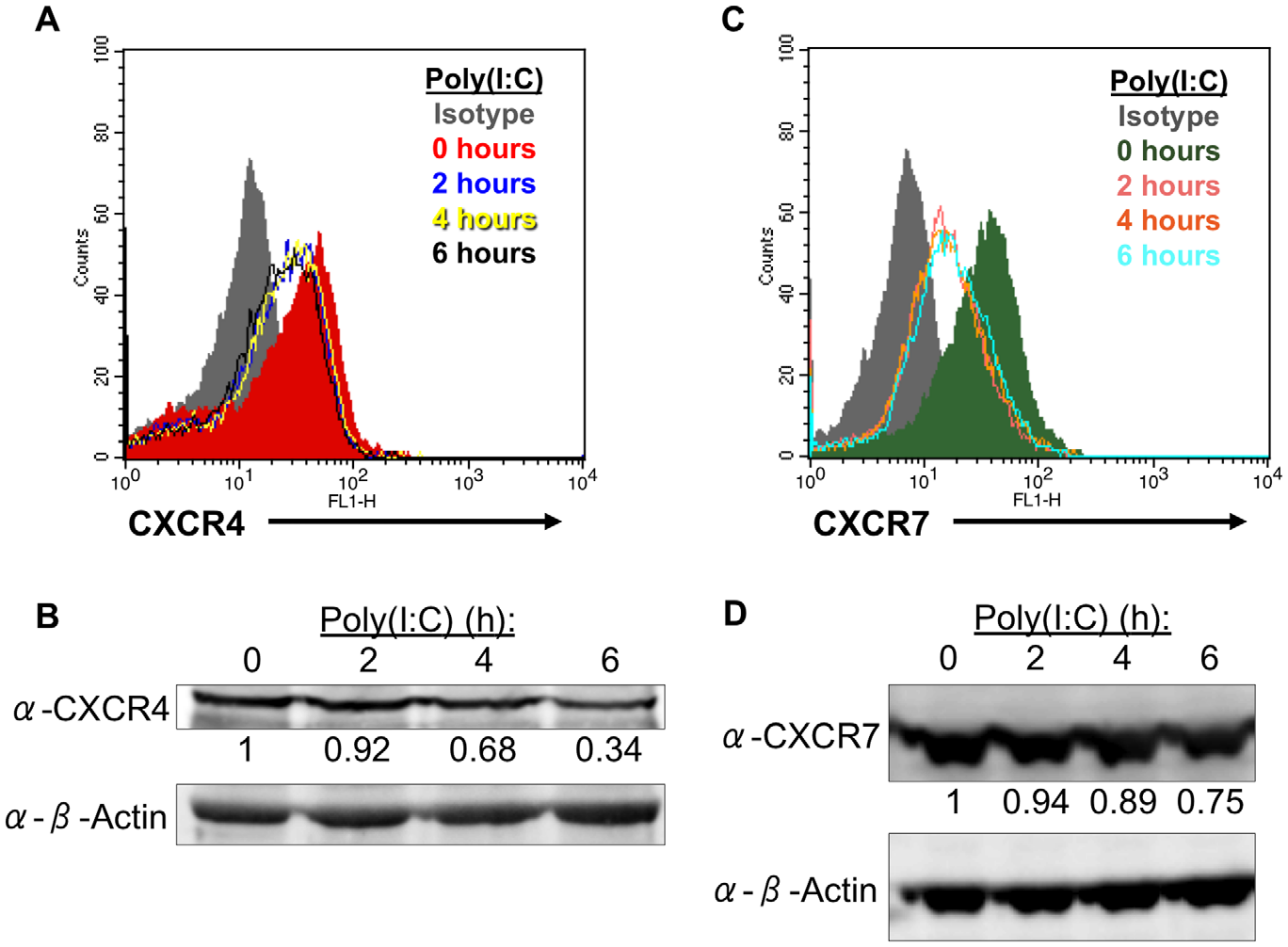
TLR3-mediated internalization and degradation of CXCR4 and CXCR7. (A, C) TLR3-stimulated, non-permeabilized hMSC were stained for cell-surface expression of CXCR4, CXCR7 or isotype control and analyzed by flow cytometry. Statistical significance was determined by comparing the experimental mean fold change in MFI to the untreated control (black, p<0.05; aqua, p<0.01). (B, D) Western blot analysis of total CXCR4 and CXCR7 expression following poly(I:C) treatment. Density values below each band are representative of results from 3 separate donors.

CXCR4 is a known mediator of hMSC migration, and a recent study found that TLR4 signaling resulted in the downregulation of CXCR4 expression in monocytes and neutrophils [34], [43], [44]. Thus, we were interested to learn if TLR3 signaling had a similar effect on CXCR4 expression in hMSC. Using flow cytometric analysis on non-permeabilized hMSC, we determined that surface expression of CXCR4 decreased starting at 2 hours and continued through 6 hours of treatment (**Figure 3A**). Statistical analysis of the fold change in mean fluorescence intensity (MFI) verified that surface expression of CXCR4 on hMSC was significantly decreased at 6 hours of treatment (black) when compared to untreated hMSC (red; p<0.01). Furthermore, Western blot analyses also indicated that total CXCR4 expression steadily decreased, suggesting this receptor is being internalized and subsequently degraded (**Figure 3B**). This internalization may not be due to CXCR4 activation as TLR3 stimulation was unable to promote any phosphorylation on serine 339 of CXCR4 (unpublished data). Therefore, this effect may likely be a TLR-mediated mechanism.

#### TLR3 activation also leads to CXCR7 internalization from the cell surface

Current reports have indicated that CXCR7, an atypical receptor also found to bind CXCL12, may modulate CXCR4 function by heterodimerizing with CXCR4 or by sequestering CXCL12 [45], [46], [47]. To date, only two studies have reported that hMSC express CXCR7, yet its function remains unknown [48], [49]. Therefore, we examined whether TLR signaling could similarly affect CXCR7 expression in hMSC as it does with CXCR4. CXCR7 was expressed on the cell surface of untreated, non-permeabilized hMSC, which substantially diminished following TLR3 activation (**Figure 3C**). Similar to CXCR4, CXCR7 surface expression was also significantly decreased after 6 hours of treatment (aqua) when compared to untreated hMSC (green; p<0.05). In addition, Western blot analyses also showed a slight decrease in total CXCR7 expression after 6 hours of treatment, indicating that CXCR7 is downregulated from the cell surface following stimulation, but is not necessarily degraded (**Figure 3D**).

### CXCR4 and CXCR7 are Internalized Following TLR3 Activation

**Figure 4:**
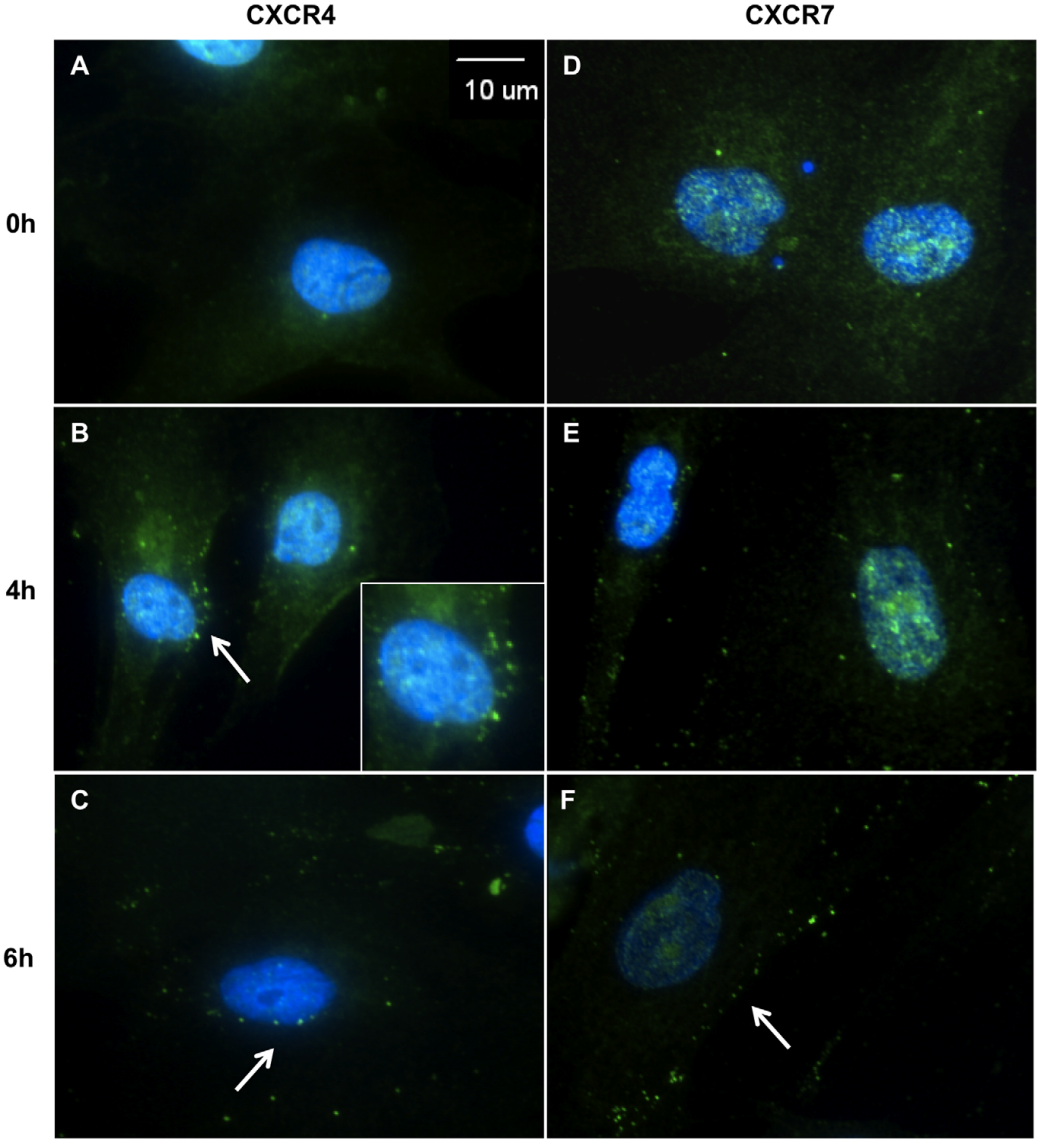
CXCR4 and CXCR7 are internalized following TLR3 activation. Human MSC were plated on chamber slides and treated for 0, 4, or 6 hours with poly(I:C) and then stained with an Alexa-488 conjugated secondary only control, Alexa-488 labeled α-CXCR4 (A-C) or α-CXCR7 (D-F) and DAPI. 40X. Scale bar represents 10 µm. Photomicrographs are representative of results from 5 separate donors.

As our flow cytometric analyses suggested that TLR3 activation results in decreased CXCR4 and CXCR7 surface expression, we then observed the cellular distribution of these chemokine receptors in hMSC. Immunofluorescence colocalization analyses indicated that CXCR4 did colocalize with CXCR7 in hMSC with a Pearson’s coefficient of 0.885; a value of 1 indicates total colocalization. The Manders’ coefficients were 0.656 for CXCR4 overlapping CXCR7 (M1), and 0.83 for CXCR7 overlapping CXCR4 (M2). As a Manders’ value of 0 indicates no colocalization and 1 indicates total colocalization, these data suggest that while only some of CXCR4 colocalizes with CXCR7, most of CXCR7 colocalizes with CXCR4 (**Figure S2F**). Furthermore, immunofluorescence staining of untreated hMSC exhibited a diffuse expression of CXCR4 and CXCR7 throughout the cytoplasm (**Figure 4A, D** **, Figure S2A-E**). However, beginning at 4 hours of treatment, both CXCR4 (**Figure 4 B, C**, arrows/insets) and CXCR7 (**Figure 4 E, F**, arrow) began to show a punctate expression pattern, an indicator of receptor internalization, which persisted through 6 hours of treatment.

### SOCS3 Modulates the Subcellular Location of CXCR4 in hMSC

**Figure 5:**
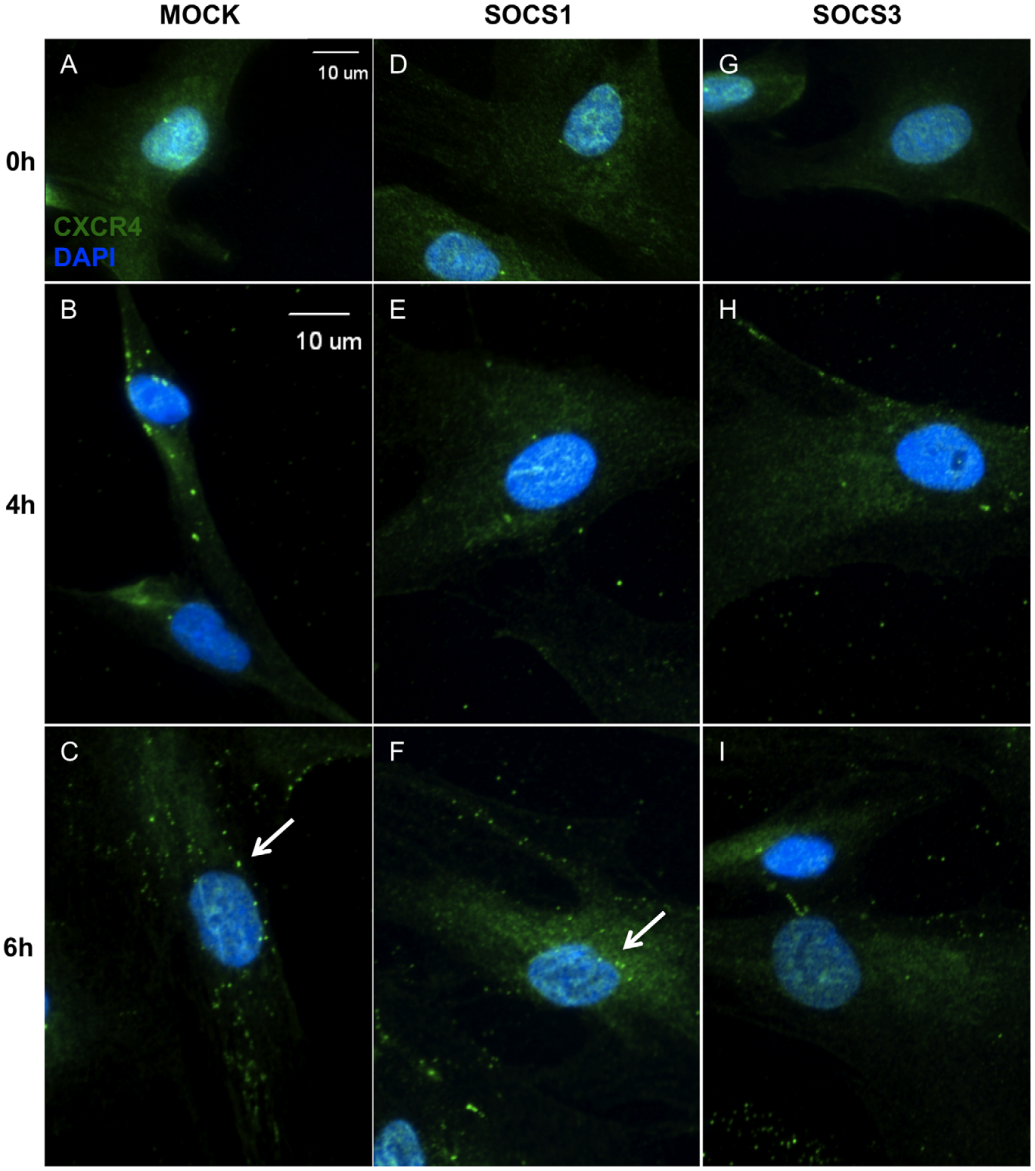
SOCS3 modulates the subcellular location of CXCR4 in hMSC. Mock-transfected (A-C), SOC1-overexpressing (D-F) or SOCS3-overexpressing (G-I) hMSC were plated onto chamber slides and treated for 0, 4, or 6 hours with poly(I:C) and then stained with an Alexa-488 conjugated secondary only control or Alexa-488 labeled α-CXCR4 and DAPI. 40X. Scale bar represents 10 µm. Photomicrographs are representative of results from 3 separate donors.

SOCS proteins have been demonstrated to inhibit CXCR4 signaling in immune cells [27]. To determine whether SOCS proteins play a role in TLR3-mediated CXCR4 internalization, we examined CXCR4 cellular distribution in hMSC overexpressing SOCS. Mock-transfected and SOCS1-overexpressing hMSC showed a CXCR4 expression pattern similar to that of untransfected cells treated with poly(I:C) (**Figure 5A-F**). However, hMSC overexpressing SOCS3 exhibited a decrease in punctate CXCR4 staining (**Figure 5H-I**).

Flow cytometry analysis of surface CXCR4 expression on SOCS-overexpressing hMSC further supports these findings. Mock-transfected and SOCS1-overexpressing, non-permeabilized hMSC treated with poly(I:C) had reduced expression of CXCR4 on the cell surface at 4 (blue) and 6 (yellow) hours of treatment (**Figure S3A**). SOCS3-overexpressing hMSC, in contrast, did not follow this trend; surface expression of CXCR4 only minimally decreased following TLR3 activation.

These finding indicate that SOCS1 impedes TLR3-mediated changes in CXCR4 expression.

### SOCS1 and SOCS3 both Modulate the Subcellular Location of CXCR7 in hMSC

**Figure 6:**
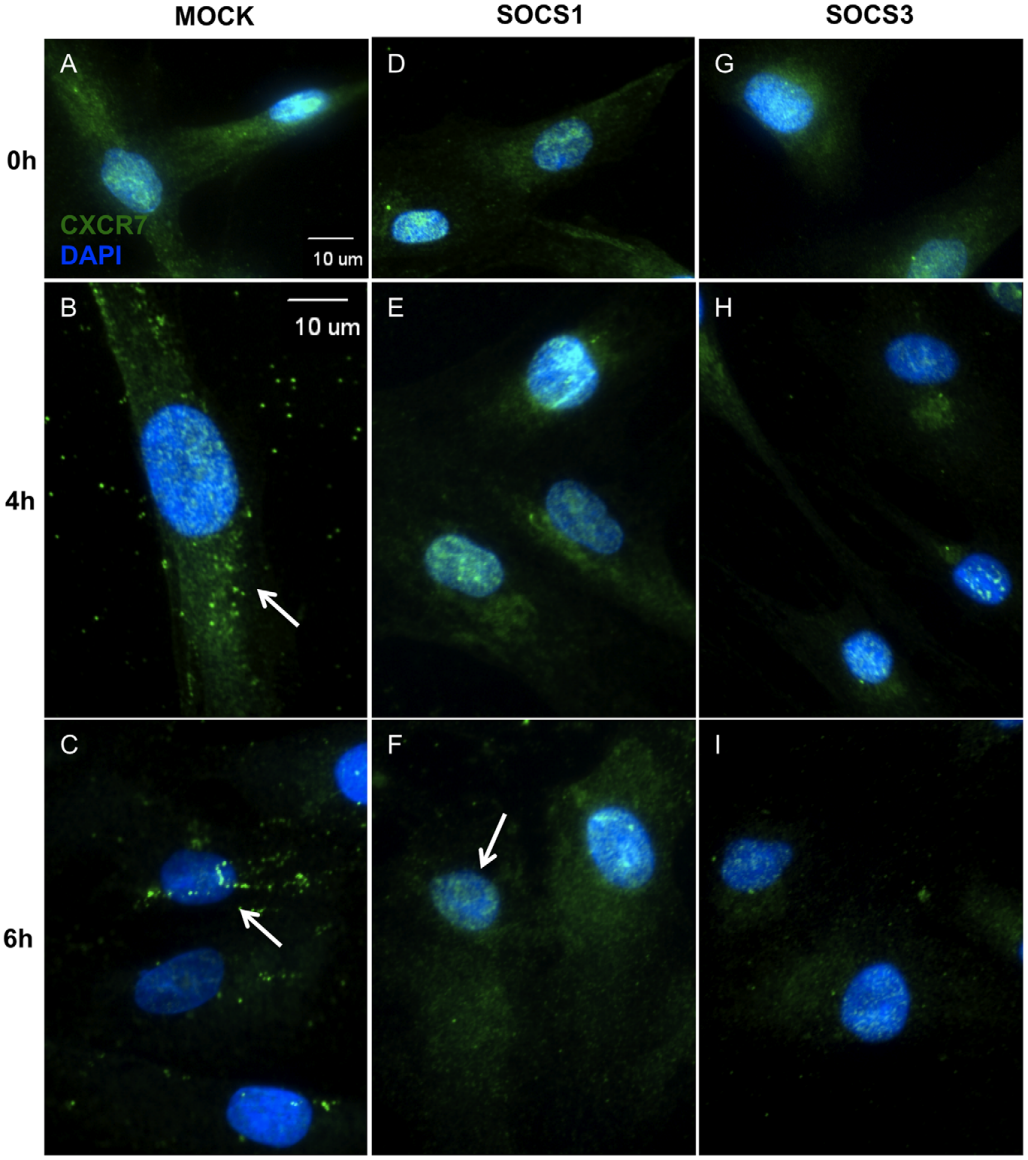
SOCS1 and SOCS3 both modulate the subcellular location of CXCR7 in hMSC. Mock-transfected (A-C), SOC1-overexpressing (D-F) or SOCS3-overexpressing (G-I) hMSC were plated onto chamber slides and treated for 0, 4, or 6 hours with poly(I:C) and then stained with an Alexa-488 conjugated secondary only control or Alexa-488 labeled α-CXCR7 and DAPI. 40X. Scale bar represents 10 µm. Photomicrographs are representative of results from 3 separate donors.

Subsequently, we also wanted to determine how SOCS might be affecting TLR3-mediated internalization of CXCR7 in hMSC. Mock-transfected hMSC showed a similar punctate staining of CXCR7 as that of untransfected hMSC following TLR3 activation (**Figure 6A-C**). In contrast, both SOCS1- and SOCS3-overexpressing hMSC showed only a diffuse cytoplasmic expression of CXCR7 following TLR3 stimulation (**Figure 6D-I**).

We also examined surface expression of CXCR7 in hMSC overexpressing SOCS following TLR3 stimulation by flow cytometry. In mock-transfected hMSC, CXCR7 expression diminished from the cell surface while hMSC overexpressing either SOCS1 or SOCS3 showed only a slight downregulation after treatment, which confirms our immunofluorescence results (**Figure S3B**). Therefore, these findings demonstrate that SOCS1 and SOCS3 are both able to disrupt TLR3-mediated CXCR7 internalization.

## Discussion

This study was prompted by the lack of publications characterizing the role of SOCS proteins in hMSC. The SOCS family of proteins are well known negative regulators of the JAK/STAT signaling pathways, and therefore serve to mediate both cytokine and chemokine signaling in leukocytes and other non-immune cells [31], [50]. Previous studies have also identified a role for SOCS proteins, mainly SOCS1 and SOCS3, as negative regulators of TLR signaling [21], [22], [23], [24], [25], [30], [51], and reports from our group and others have determined that TLRs are expressed and functional in MSC [15], [16], [17], [18], [19]. While research is currently underway investigating the role of TLRs in MSC biological processes, including proliferation, differentiation, migration, and immunomodulation [15], [16], [17], [18], [19], there were no known studies exploring SOCS and MSC function.

**Figure 7:**
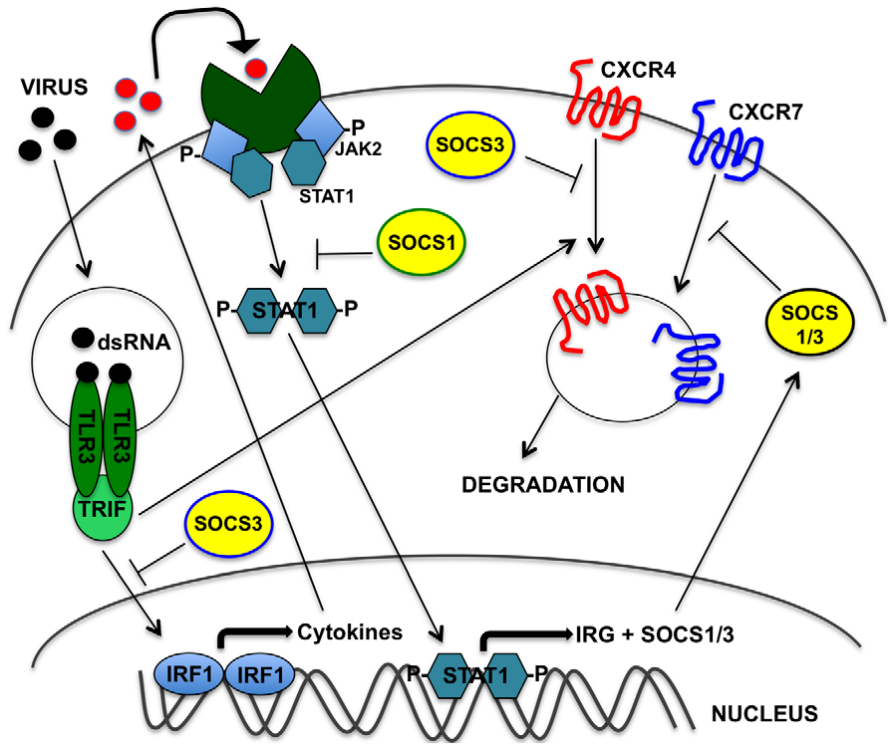
SOCS1 and SOCS3 inhibit TLR3 signaling in hMSC. TLR3 signaling induces cytokine expression and mediates the internalization of both CXCR4 and CXCR7, as well as the degradation of CXCR4. A JAK2/STAT1 pathway is also activated, and subsequent upregulation of SOCS1 and SOCS3 inhibits TLR3-mediated signaling by targeting different branches of the signaling pathways. SOCS1 inhibits STAT1 phosphorylation and CXCR7 internalization, while SOCS3 inhibits IRF1 upregulation and the internalization of both CXCR4 and CXCR7.

This study established that in TLR3-stimulated hMSC, the transcription factor, IRF1 was upregulated, and a JAK2/STAT1 downstream pathway was indirectly activated following an induction of a unique cytokine expression profile, which we have previously described [17], [19]. Furthermore, both SOCS1 and SOCS3 expression was upregulated after TLR3 signaling. Our subsequent overexpression studies identified that SOCS1 and SOCS3 each play a distinct role in negatively regulating TLR3 and JAK/STAT signaling pathways, where SOCS1 specifically inhibited STAT1 activation, while SOCS3 inhibited IRF1 upregulation. Furthermore, these results collectively demonstrate that SOCS1 and SOCS3 play a distinct role in modulating TLR3 and JAK/STAT signaling in hMSC, and have been incorporated into a proposed signaling model (**Figure 7**).

In addition to these findings, we also identified a unique chemokine receptor response in TLR3-stimulated hMSC. We found that in TLR3-activated hMSC, CXCR4 expression was internalized and degraded. Furthermore, we also determined that TLR3 stimulation induced the internalization of CXCR7. We propose that the removal of CXCR4 and CXCR7 by internalization and/or degradation following TLR3 activation may serve as one mechanism to prevent signaling of these receptors, while enhancing its ability to migrate towards a TLR ligand. In support of this mechanism, we have previously shown that migration assays using hMSC primed with TLR ligands show an enhanced migratory ability towards a serum chemoattractant, but only when pretreated for one hour [19]. In contrast, we found that hMSC that were TLR3-primed for 24 hours showed a severely decreased migratory capacity towards a serum chemoattractant. Since CXCR4 is a well-known mediator of hMSC migration and serum is known to contain its ligand, CXCL12, we might speculate that one reason we see a decreased migration capacity towards a serum chemoattractant following 24 hour TLR3 priming, is because this priming causes the downregulation of CXCR4 from the hMSC cell surface [34], [44].

On a broader scale, by inhibiting CXCR4 signaling through internalization, TLR-activated hMSC may no longer be retained in the bone marrow by the presence of CXCL12, and may migrate out of the bone marrow to sites of stress which secrete factors, or danger signals, recognized by TLRs. Additionally, TLR signaling may permit hMSC to efficiently migrate to a site of stress, by temporarily depleting these receptors from the cell, thus inhibiting retention in the bone marrow while also preventing its migration toward other chemoattractants. Once they have reached their targeted site, hMSC may then fulfill their immunomodulatory and reparative functions.

Previous reports indicate that SOCS proteins also negatively regulate chemokine signaling by docking to a target receptor, thus inhibiting both signaling and cell function, such as migration [26], [27]. Our study found that TLR3-mediated internalization of CXCR4 and CXCR7 was inhibited in hMSC overexpressing SOCS. Flow cytometry and immunofluorescentce studies indicate SOCS3 overexpression inhibited receptor internalization of CXCR4 while both SOCS1 and SOCS3 overexpression prevented CXCR7 receptor internalization. These results suggest that SOCS1 and SOCS3 may be modulating the TLR3-mediated mechanism of CXCR4 and CXCR7 downregulation, and not necessarily directly inhibiting the receptors themselves. If SOCS were inhibiting these chemokine receptors, we would expect to see a punctate staining pattern of these receptors in untreated, SOCS-overexpressing hMSC.

Lastly, the signaling scheme we identified here downstream from short-term stimulation of TLR3 to IFN→ JAK/STAT →SOCS 1/3→ CXCR4/7 that drives the migration of the hMSC appears to be even more complex than initially anticipated. Indeed, we were not able to demonstrate a direct effect on hMSC migration after specific inhibition of any one of the downstream molecules by transwell, wound, or Boyden assays. This implies that rather than linear signaling pathways there must be various redundant pathways involved that mediate hMSC migration. Further investigations are necessary of the TLR-mediated down-regulation of CXCR4, CXCR7 and potentially other chemokine receptors in hMSC after TLR stimulation. This information is essential to improving hMSC-based therapy that enhances the mobilization of the MSCs to the targeted sites where they will be able to exert their established immunomodulatory and reparative functions.

## Supporting Information

Figure S1 **TLR3 signaling activates neither STAT3 nor STAT5.** Phosphorylation levels of STAT3 (A) and STAT5 (B) in hMSC following TLR3 activation was determined by Western blot analysis. Densitometry was determined by subtracting overall background, then each experimental band was normalized to the actin loading control band within its lane, and fold change was calculated based upon the untreated control band. Density values below each band are representative of results from 3 separate donors.
(TIF)

Figure S2 **CXCR4 and CXCR7 are colocalized in hMSC.** Human MSC were plated onto chamber slides and stained with an Alexa-568 and Alexa-488 conjugated secondary only control or an Alexa-568 labeled α-CXCR4 (red) and Alexa-488 labeled α-CXCR7 (green) and DAPI (blue). 40X. Scale bar represents 10 µm. Photomicrographs are representative of results from 3 separate donors. Both Manders’ and Pearson’s coefficients were used to determine colocalization.
(TIF)

Figure S3 **SOCS inhibits TLR3-mediated internalization of CXCR4 and CXCR7.** Human MSC that were mock-transfected, or overexpressing either SOCS1 or SOCS3 were stained for cell-surface expression of CXCR4 (A), CXCR7 (B) or isotype control following TLR3 stimulation and analyzed by flow cytometry. Histograms are representative of results from 3 separate donors.
(TIF)
