# Supplementary figures and images for: Toll-Like Receptor 3 and Suppressor of Cytokine Signaling Proteins Regulate CXCR4 and CXCR7 Expression in Bone Marrow-Derived Human Multipotent Stromal Cells
Source: PLoS One. 2012 Jun 22;7(6):e39592. doi: 10.1371/journal.pone.0039592 (PMC3382127; doi:10.1371/journal.pone.0039592)

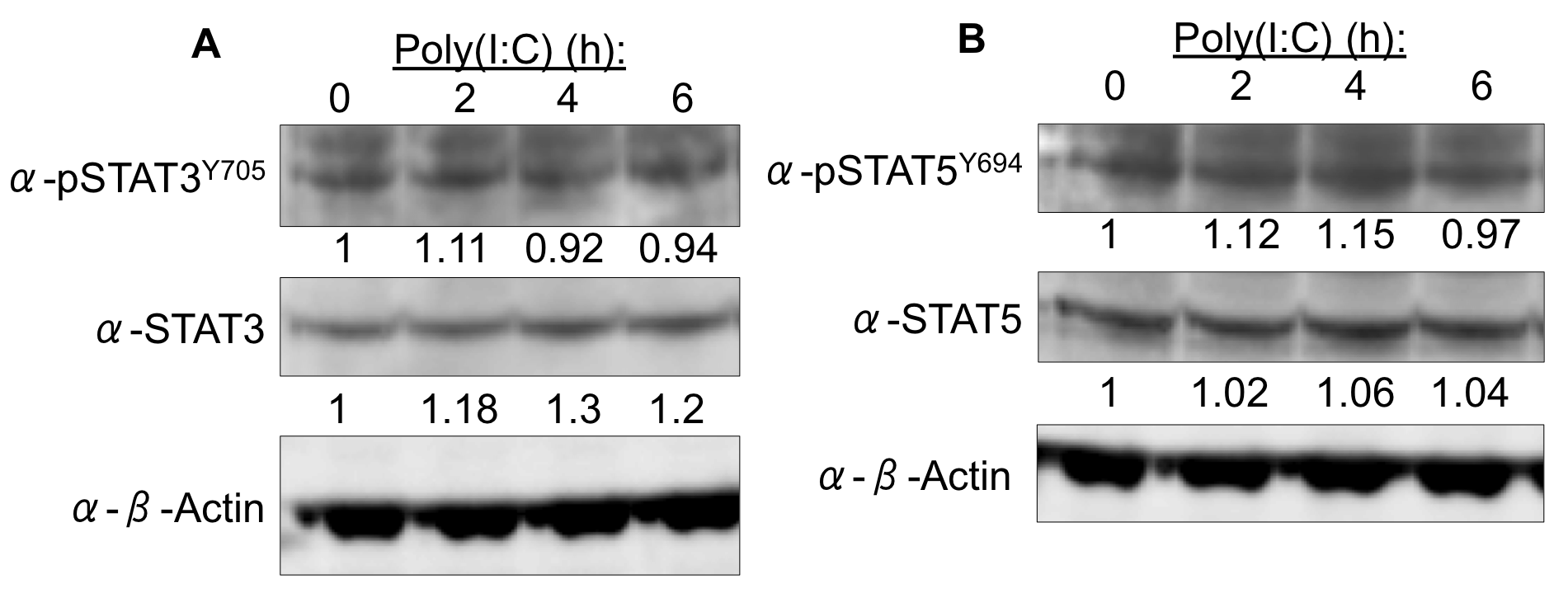

Supplement: Figure S1 — TLR3 signaling activates neither STAT3 nor STAT5. Phosphorylation levels of STAT3 (A) and STAT5 (B) in hMSC following TLR3 activation was determined by Western blot analysis. Densitometry was determined by subtracting overall background, then each experimental band was normalized to the actin loading control band within its lane, and fold change was calculated based upon the untreated control band. Density values below each band are representative of results from 3 separate donors. (TIF) [file pone.0039592.s001.tif]

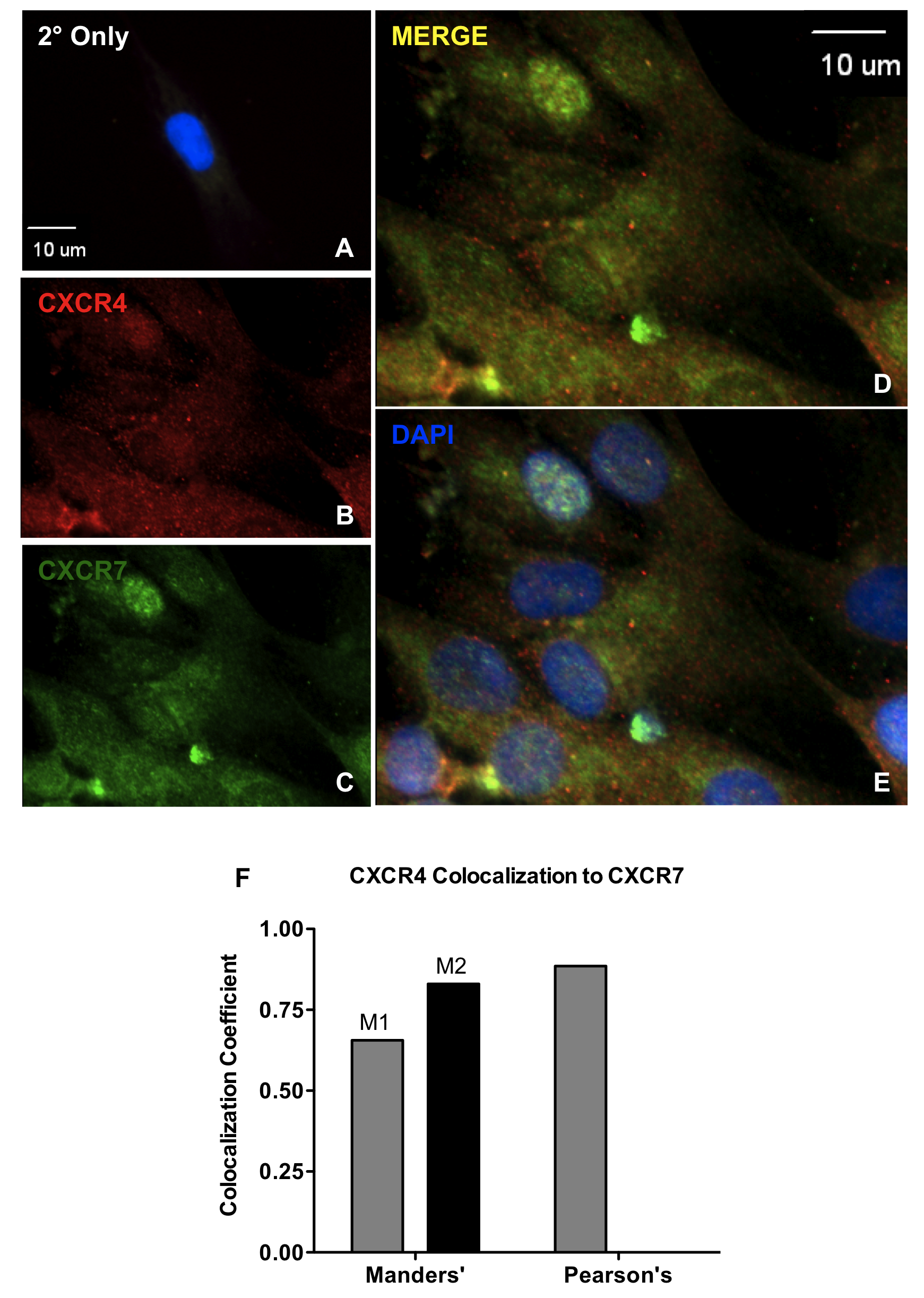

Supplement: Figure S2 — CXCR4 and CXCR7 are colocalized in hMSC. Human MSC were plated onto chamber slides and stained with an Alexa-568 and Alexa-488 conjugated secondary only control or an Alexa-568 labeled α-CXCR4 (red) and Alexa-488 labeled α-CXCR7 (green) and DAPI (blue). 40X. Scale bar represents 10 µm. Photomicrographs are representative of results from 3 separate donors. Both Manders’ and Pearson’s coefficients were used to determine colocalization. (TIF) [file pone.0039592.s002.tif]

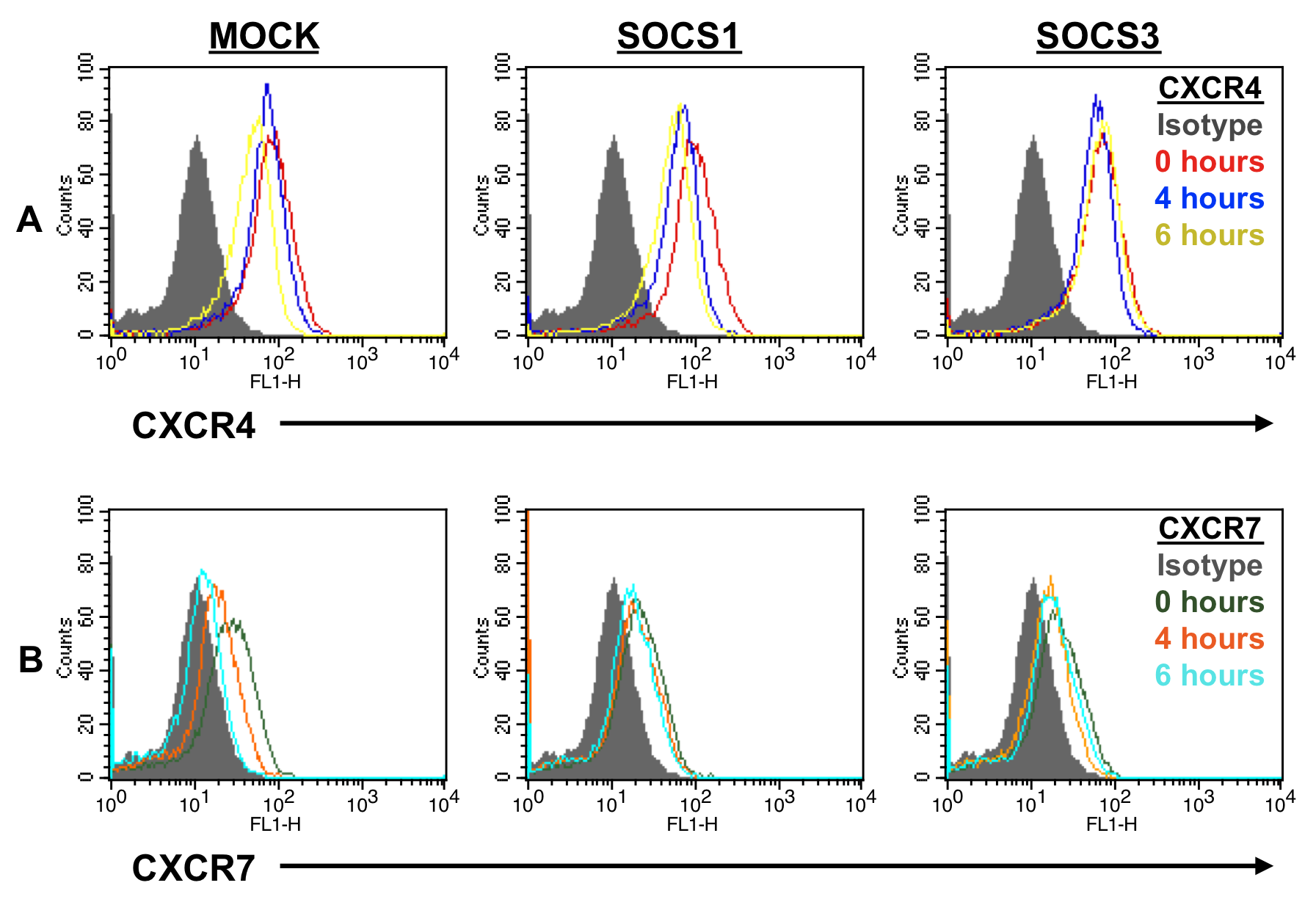

Supplement: Figure S3 — SOCS inhibits TLR3-mediated internalization of CXCR4 and CXCR7. Human MSC that were mock-transfected, or overexpressing either SOCS1 or SOCS3 were stained for cell-surface expression of CXCR4 (A), CXCR7 (B) or isotype control following TLR3 stimulation and analyzed by flow cytometry. Histograms are representative of results from 3 separate donors. (TIF) [file pone.0039592.s003.tif]
